# Cannabinoids and Prostate Cancer: A Systematic Review of Animal Studies

**DOI:** 10.3390/ijms21176265

**Published:** 2020-08-29

**Authors:** Kanika Singh, Negar Jamshidi, Roby Zomer, Terrence J. Piva, Nitin Mantri

**Affiliations:** 1 The Pangenomics Lab, School of Science, RMIT University, Bundoora, Victoria 3083, Australia; kanika.singh@rmit.edu.au; 2 School of Health and Biomedical Sciences, RMIT University, Bundoora, Victoria 3083, Australia; negar.jamshidi@rmit.edu.au (N.J.); terry.piva@rmit.edu.au (T.J.P.); 3 MGC Pharmaceuticals Limited, West Perth, Western Australia 6005, Australia; roby@mgcpharma.com.au

**Keywords:** animal models, cancer, cannabinoids, cannabis, WIN55,212-2, prostate cancer

## Abstract

Prostate cancer is a major cause of death among men worldwide. Recent preclinical evidence implicates cannabinoids as powerful regulators of cell growth and differentiation, as well as potential anti-cancer agents. The aim of this review was to evaluate the effect of cannabinoids on in vivo prostate cancer models. The databases searched included PubMed, Embase, Scopus, and Web of Science from inception to August 2020. Articles reporting on the effect of cannabinoids on prostate cancer were deemed eligible. We identified six studies that were all found to be based on in vivo/xenograft animal models. Results: In PC3 and DU145 xenografts, WIN55,212-2 reduced cell proliferation in a dose-dependent manner. Furthermore, in LNCaP xenografts, WIN55,212-2 reduced cell proliferation by 66–69%. PM49, which is a synthetic cannabinoid quinone, was also found to result in a significant inhibition of tumor growth of up to 90% in xenograft models of LNCaP and 40% in xenograft models of PC3 cells, respectively. All studies have reported that the treatment of prostate cancers in in vivo/xenograft models with various cannabinoids decreased the size of the tumor, the outcomes of which depended on the dose and length of treatment. Within the limitation of these identified studies, cannabinoids were shown to reduce the size of prostate cancer tumors in animal models. However, further well-designed and controlled animal studies are warranted to confirm these findings.

## 1. Introduction

Currently, prostate cancer accounts for 14% of all malignancies in males and is second to lung cancer as the leading cause of death across 46 countries. It has the fifth highest mortality rate (6.7%) worldwide [1]. Despite improved diagnostic technology, with nearly 1.3 million new cases diagnosed in 2018, approximately 359,000 deaths associated with prostate cancer were reported [1]. While the etiological factors for prostate cancer are not fully understood, there are a number of factors associated with the risk of developing the disease, such as age, family history, lifestyle-related factors (e.g., smoking and diet), and testosterone levels [2].

The most commonly mutated genes observed in primary prostate cancers include SPOP, TP53, FOXA1, and PTEN [3]. The retinoblastoma tumor suppressor gene RB1 is more commonly mutated in metastatic and ADT-recurrent prostate cancer than in primary tumors, while the expression of the ST6GalNAc1 gene is upregulated in primary prostate cancer cells and repressed in patients undergoing androgen deprivation therapy [4]. The prostate specific antigen (PSA)—an androgen receptor which is widely employed as a marker in the detection of early prostate cancer—is regulated by androgens [5]. Increased PSA levels are used as a biomarker of prostate disorders, including prostate cancer, prostatitis, and benign prostatic hypertrophy [6]. In LNCaP prostate cancer cells, androgens work through the androgen receptor to regulate both PSA mRNA and glycoprotein levels [7]. Sharma et al. [6] observed that cannabis extracts reduced both intracellular PSA mRNA expression and secreted PSA levels, implying that cannabinoid receptor agonists may be exploited to prevent prostate cancer progression.

Prostate cancer can be treated by either conventional or alternative treatment methods. Conventional strategies for treating localized prostate cancer are determined by the patient’s age, condition, and preferences of available treatment regimens. The introduction of targeted therapy has been a major success in cancer treatment in the past few decades. In cases of metastatic prostate cancer, radical treatment with a curative intent is recommended. Radical treatments may result in significant adverse events, including sexual dysfunction, bone fractures, diabetes, cardiovascular morbidity, acute myocardial infarction, or dementia [8]. Taking into consideration such adverse events, there is an urgent need for the development of safer and more effective treatment therapies.

Plants have been used in traditional medicines for the treatment of numerous ailments, such as cancer, cardiovascular, inflammatory, metabolic, parasitic, and viral diseases [9]. Recently, attention has focused on alternative therapies that offer fewer side effects compared to the conventional treatment modalities. Traditionally, prostate cancer treatment includes the use of traditional Chinese medicine [10], such as Celastrol, which is an active compound extracted from the root bark of *Tripterygium wilfordii*, commonly known as “Thunder of God Vine” [11,12]; *Ginkgo biloba* [13]; *Dysosma versipellis* [14]; *Saussurea involucrate* [15]; and other traditional plants, including *Cannabis sativa* [6,16,17,18]. Cannabis has always been very controversial due to its recreational uses; however, in recent years, an increasing public and scientific interest in its medical applications has emerged [19]. The major active components of cannabis are tetrahydrocannabinol (THC) and cannabidiol (CBD)—more commonly known as cannabinoids [20,21].

Recent evidence suggests that cannabinoids are powerful regulators of cell growth and differentiation [22]. They have demonstrated anti-tumor effects in experimental models by decreasing the viability, proliferation, adhesion, and migration of various cancer cells [23,24,25]. Therefore, cannabinoids can be potentially used in the treatment of prostate, glioma, and breast cancers, as well as immune-related malignancies [24,26,27]. One of the advantages of using medical cannabis is that it specifically targets the tumor cells, but has a low potency towards non-tumor cells. This is of significant importance when compared to chemotherapy, where non-tumor cells are also affected by the cytotoxic effects of these agents [6,22,28].

The anticancer activity of cannabinoids, such as the inhibition of prolactin-induced proliferation, epidermal growth factor (EGF)-induced proliferation, and androgen-independent cancer cell invasion, frequently occurs due to various mechanisms, such as the inhibition of prolactin receptor expression, blocking cells at the G1/S checkpoint, and downregulating the production of EGF [29]. There are two main cannabinoid receptors (CB1 and CB2) found on the cell membrane and they possess 44% homology [30]. Both cannabinoid receptors are G-protein-coupled receptors [31] and contain seven transmembrane domains, along with an intracellular C-terminal and extracellular N-terminal domain [32]. The expression of cannabinoid receptors on the surface of prostate cancer cells is greater than that seen in non-cancerous cells, which suggests that the endocannabinoid system may play a crucial role in the growth of these cancer cells [33]. Other have also suggested that CB1 and CB2 play a role in the development of prostate cancers [29,34,35,36].

Recent studies have highlighted the role that CB2 receptors play in regulating tumor cell metastasis [37]. Cannabinoid receptors are known to regulate the phosphorylation and activation of various members of the family of mitogen-activated-protein kinases (MAPKs), including extracellular signal regulated kinase-1 and -2 (ERK1/2), p38 MAPK (p38), and c-Jun n-terminal kinase (JNK) [38]. The MAPK pathway controls gene expression related to cell proliferation, motility, adhesion, and apoptosis, as well as that of glucose metabolism [39]. Morell et al. [33] reported that the cannabinoid WIN55,212-2 inhibited the PI3K/AKT/mTOR signaling pathway in neuro-endocrine differentiated prostate cancer cells. WIN55,212-2 prevented the stimulation of the AMP-activated protein kinase (AMPK) signaling system in LNCaP cells [33], which modulated their proliferation and survival [40].

It has been shown that the effects exerted by cannabinoids are cell line- or tumor type-dependent [41]. Orellana-Serradell et al. [42] reported that endocannabinoids inhibited the growth of PC3 prostate cancer cells via inhibiting adenylate cyclase and protein kinase A activity, and arrested the cell cycle via the induction of p27 and downregulation of the EGF receptor. The growth of primary cultures of prostate tumors was inhibited by endocannabinoids, which triggered these cells to undergo apoptosis, probably through activation of the ERK signaling pathway [42].

Furthermore, while a large number of in vitro studies have provided evidence of positive outcomes when using cannabinoids on prostate cancer cells [6,43,44], there are relatively very few in vivo studies reported in the literature to date [45,46]. In addition, there are no published clinical studies on cannabis use in prostate cancer, despite extensive experimental in vitro studies highlighting their effectiveness [42,47]. Most studies on the potential therapeutic use of cannabis and cannabinoids address their efficacy in relieving the symptoms of cancer and of treatments such as chemotherapy [23,48]. In this systematic review, we evaluate the role of cannabis in the treatment of prostate cancer in animal models.

## 2. Results

### 2.1. Results from the Search

**Figure 1:**
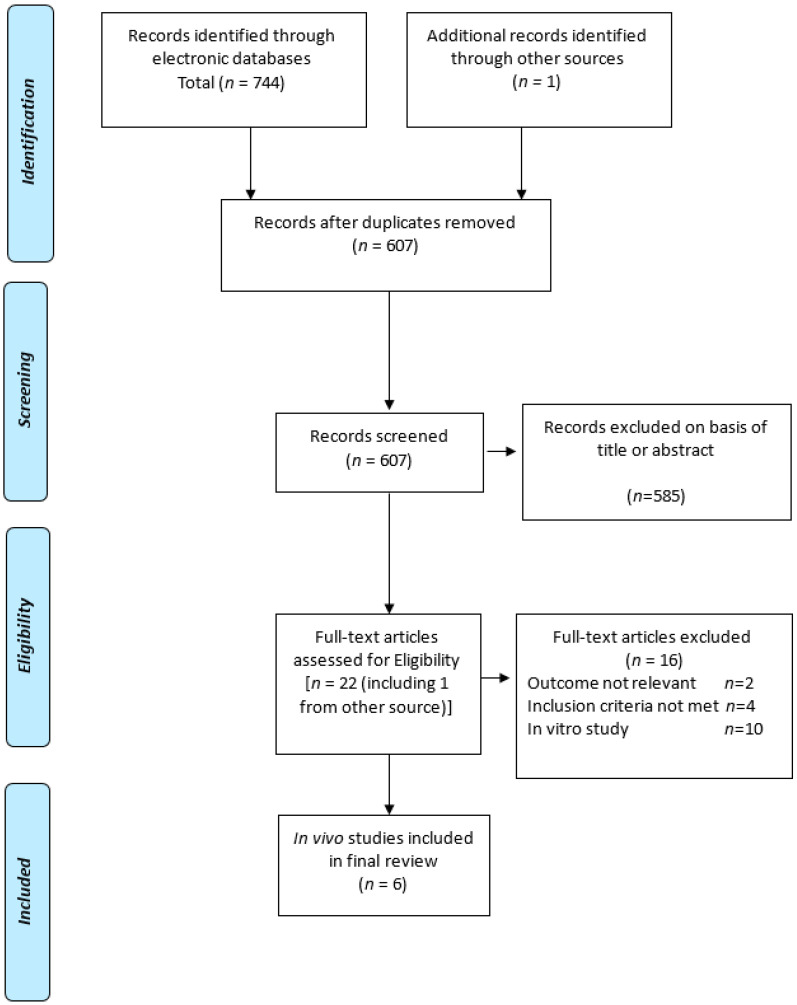
Preferred Reporting Items for Systematic Reviews and Meta-Analyses (PRISMA) flow chart.

Following an extensive search, a total of 307 unique studies after deduplication were included. Through screening based on the title and abstract, 278 studies were excluded. At the full text screening stage, a further 23 potentially relevant studies were excluded, resulting in a final total of six studies deemed eligible for inclusion in this systematic review. The flow chart of the study conducted according to Preferred Reporting Items for Systematic Reviews and Meta-Analyses (PRISMA) is summarized in Figure 1.

### 2.2. Risk of Bias Assessment

**Figure 2:**
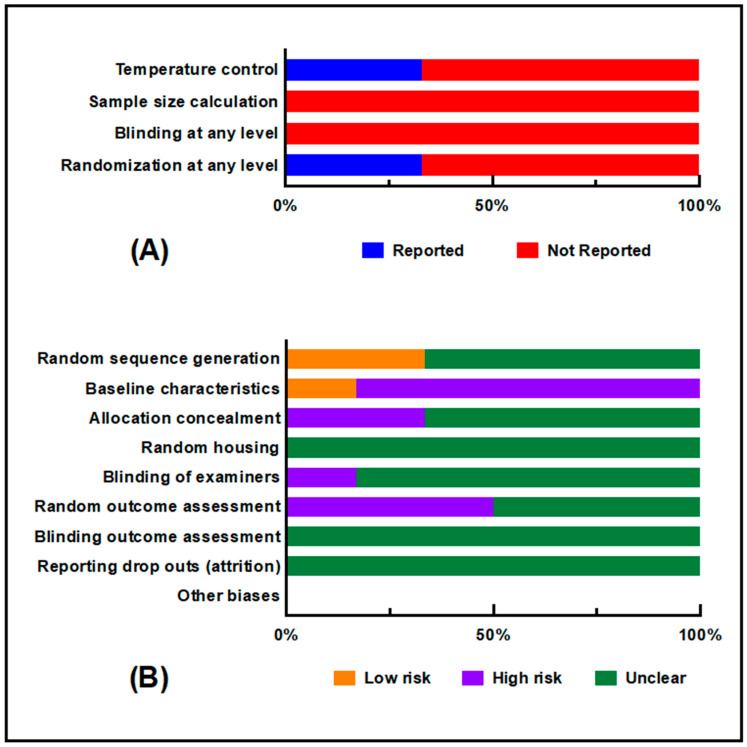
Risk of bias and quality assessments. (**A**) Quality indicators; (**B**) Risk of bias assessment according to each of the SYRCLE criteria.

The results of the risk of bias [49] assessment in these studies are presented in Table 1 (individual scores) and Figure 2. Randomization was only reported in two studies (33.3%) [33,46], but it was unclear whether this occurred in the other studies. Similarly, temperature controls were only reported in two studies (33.3%) [33,45]. No information on blinding or size calculations was reported in any study (Figure 2A). Therefore, the risk of bias due to random housing and blinding of the assessor was unclear. The risk of bias due to the concealment of group allocation during the experiment was assessed as high for two studies, but was unclear for the others [33,45]. A high risk of bias was assessed as insufficient information was reported for the baseline group characteristics in five out of the six studies. No information was provided regarding attrition bias in these papers. Random group allocation was reported for two studies, but was not clear in the others [33,46]. The blinding of examiners was high in one study, but unclear in the others [45].

### 2.3. Study Characteristics

The characteristics of the six studies are summarized in Table 2. These in vivo studies were performed on different male mice strains [45,46]. These included athymic nude mice aged between 4 and 7 weeks and athymic nude-Foxn1 (nu/nu), MF-1 nude mice, and BALB/cOlaHsd-Foxn1nu nude mice. Human prostate tumor cell lines LNCaP, DU145, PC3, and CWR22Rv1 were used to generate tumors in these animals. The tumors were induced in the flank of the mice via subcutaneous injection [33,45,46]. Mice were injected with a minimum of 1 × 10^6^ prostate cancer cells to a maximum of 1–2 × 10^7^ prostate cancer cells, in order to establish the tumor in reported studies. In Olea-Herrero’s study, mice were injected with 2 × 10^6^ PC3 cells [50]; in Mukhtar’s study, the mice were injected with 1 × 10^6^ LNCaP cell [51]; in Morell’s study, mice were injected with 5 × 10^6^ PC3 cells [33]; in De Petrocellis’ study, 1–2 × 10^7^ LNCaP or DU145 cells were injected into the mice [45,52]; in Roberto’s study, 1 × 10^6^ PC3 cells were injected into the mice [46]; and in the study by Morales, it is mentioned that the mice were injected with LNCaP and PC3 cancer cells subcutaneously [52].

Cannabinoid treatment was initiated once the required tumor volume was between 70 and 150 mm^3^ in these subcutaneous xenografts [33,46,50,51,52]. These tumors were treated with a range of cannabinoids, including WIN55,212-2, CBD-BDS (biological drug substance) plus Docetaxel, CBD-BDS plus bicalutamide, JWH015, JWH015 plus SA2 (specific antagonist), and Chromenopyrazoledione 4 (PM49, a synthetic cannabinoid). The duration of treatment varied from 14 to 38 days. Cannabinoids were administered orally, intraperitoneally, subcutaneously, or intravenously at doses between 0.5 and 100 mg/kg body weight.

## 3. Discussion

This systematic review highlights six studies reporting the effectiveness of both synthetic and phytocannabinoids in in vivo/xenograft models for prostate cancer. In this review, we have gathered detailed information on the study population, group size, cell lines that were used for inoculation and tumor generation, intervention that was used for treatment (natural/synthetic cannabinoid), dose size, route of administration, duration of the study, and reduction of the tumor size (Table 2). Two earlier systematic reviews [45,53,54] briefly explored the effect of cannabis in experimental models of prostate cancer, such as in vitro studies [42,47] and urological tumors [53,54].

Recently, cannabinoids have been shown to inhibit cell proliferation, migration, and angiogenesis, as well as arrest the cell cycle and induce apoptosis in prostate cancer cells [46]. Furthermore, a number of in vitro studies have reported that synthetic cannabinoids such as WIN55,212, JWH-133, and JWH-015 can reduce the size of prostate cancer cell-derived tumors [55,56]. Of the six papers examined, five used synthetic cannabinoids [33,46,50,51,52], while the other used natural cannabinoids [45]. Most studies demonstrated a reduction in tumor size post-cannabinoid administration. In each of these studies, there was a minimum of eight mice/treatment group. The minimum dose required to start the tumor was 1 × 10^6^ prostate cancer cells (PC3, DU145, or LNCaP), which was injected into either the left or right flank of the mice. All six studies reported that the prostate cancer cells were injected subcutaneously into mice to induce tumors.

Once the tumors had established themselves, the cannabinoids were administered intraperitoneally, as seen in four of the six studies [33,45,51,52]. Englund et al. [57] stated that when the phytocannabinoid THC was administered intravenously to the human participants, they showed different psychosomatic symptoms, such as anxiety, hallucinations, psychotomimetic effects, a blunted effect, paranoia, conceptual disorganization, illusions, depersonalization, slowing of time, emotional withdrawal, lack of spontaneity, and many more. However, when participants were given CBD intravenously, they did not feel any of the above symptoms and no side effects were reported. No comment was made on any behavioral changes in the animals receiving treatment.

Among the included studies, the synthetic cannabinoid agonist WIN55,212-2 was used in three studies and was shown to inhibit tumor growth [33,46,51]. Roberto et al. [46] observed that the synthetic cannabinoid WIN55,212-2 reduced the size of PC3-, DU145-, and LNCaP-induced tumors by 46–69%, and this reduction in size was dose dependent. Morell et al. [33] did not report the actual percentage of reduction in tumor size; however, they reported treating athymic mice for 15 days with a dose of 0.5 mg/kg WIN55,212-2, and noted that the WIN55,212-2-treated xenografts were smaller in size compared to the untreated controls. Correspondingly, βIII Tub levels (a neuroendocrine marker expressed in cancer cells, such as prostate cancer, non-small-cell lung carcinoma, and breast and ovarian cancer [58]) were lower in the WIN55,212-2-treated tumors compared to the untreated tumors. Additionally, Morales et al. [52] demonstrated that, when LNCaP and PC3 xenografts were treated with 2 mg/kg of PM49 (the most potent derivative of the synthetic cannabinoid quinone), treatment almost totally blocked the growth of LNCaP tumors, whereas it inhibited the growth of PC3 tumors by 40%. They also stated that treatment with PM49 was more effective in LNCaP xenografts and is androgen sensitive, unlike that of PC3 [52]. However, Olea-Herrero et al. [50] reported a reduction in tumor size, but did not quantify the difference.

In the study by De Petrocellis et al. [45], a phytocannabinoid CBD-botanical drug substance was used as the active compound. In this study, CBD was used in combination with anticancer agents, such as Docetaxel and Bicalutamide, which effectively inhibited tumor growth. Similarly, Scott et al. [59] studied combinations of the cannabinoids CBD, cannabigerol (CBG), and cannabigevarin (CBGV) in their neutral forms in leukemia cells. They demonstrated that CBD acts non-antagonistically with other cannabinoids to reduce the cell number and that the cannabinoid activity is influenced by the drug combination and treatment schedule. Other studies have also highlighted the toxicity of cannabinoids on tumor cells [60,61,62]. Müller et al. [60] showed that WIN55,212-2 caused a significant dose-dependent effect on the viability of A549 lung cancer cells, HoTu-10 testicular cancer cells, and IMR-5 neuroblastoma cells. Casanova [61] has also reported similar outcomes, showing that WIN55,212-2 and JWH-133 caused a 75% reduction of skin tumor growth in vivo. Finally, Sanchez et al. [62] observed that the synthetic cannabinoid JWH-133 significantly inhibited the proliferation of brain tumors compared to untreated controls.

In particular, emerging evidence suggests that cannabinoids have a dual role in counteracting prostate cancer progression, as well as the proliferation of stromal cells in the prostate tumor microenvironment. In a recent study, Pietrovito et al. [36] reported that the cannabinoid treatment of prostate cancer cells (LNCaP, PC3, and DU145) selectively impaired cell-survival, while at the same time regulating prostrate stromal fibroblast phenotypes under in vitro conditions. The authors further showed that the activity of the synthetic cannabinoid WIN 55-212-2 was mediated by the increased expression of CB2 receptors, which are normally downregulated in healthy prostate fibroblast cells. The expression of both CB1 and CB2 receptors was elevated in LNCaP cells compared to PC3 and DU145 cells [36]. Similar results were also found by Roberto et al. [46], who demonstrated that WIN55,212-2 substantially reduced cell proliferation, invasion, and migration, as well as inducing G0/G1 cell cycle arrest apoptosis, in a dose-dependent manner in cultured PC3, DU145, and LNCaP prostate cancer cells. These effects were mediated through a pathway involving the cell cycle regulators p27, Cdk4, and pRb [46]. Collectively, the evidence from in vitro and in vivo studies highlights the anti-cancer characteristics of phyto-, endo-, and synthetic cannabinoids in prostate cancer.

Interestingly in recent years, cannabinoids have been extensively studied for their potential anticancer effects, as well as for symptomatic management in cancer patients. They are known to interact with the components of the endocannabinoid system or other cellular pathways and thus affect both tumor development and progression. Cannabidiol has been shown to exert chemo-preventive effects in preclinical models of prostate cancer [63]. In a recent clinical trial, Kenyon et al. [64] reported that an initial dose of 10 drops (10 mg) twice a day of cannabinoids (three days on and three days off) reduced the numbers of circulating prostate tumor cells when compared to the effects elicited with cannabidiol.

When evaluating in vivo studies, an important aspect is to identify bias that might be present and ways to reduce it, if possible. For selection bias, it was not clear based on which baseline grouping characteristics the authors grouped animals and if the identity of the allocated animals was concealed. The performance bias item random housing of animals was not reported adequately and thus represents a potential risk of bias. The blinding of investigators, as well as risk of bias due to dropout/attrition, were also not reported in the included studies.

This systematic review has some limitations. The searches were only conducted in PubMed, Scopus, Web of Science, and Embase. While unlikely, it is possible that additional articles/information would have been discovered had other databases been included, and if the search strategy had included gray literature resources, dissertations and theses, conference proceedings, and non-English language articles. Due to insufficient in vivo evidence, further comprehensive in vivo studies are required to fully understand the synergistic effect and molecular pathways that lead to anticancer effects of combinatorial therapies, such as cannabidiol and DNA-damaging agents (temozolomide, or cisplatin) [65]. Although cannabinoids may potentially assist with the management of prostate cancer, there is still a pressing need to identify the most effective combination(s) of drugs for the treatment of prostate cancer, as well as other cancers.

In the past decade, extensive research has been undertaken to identify the therapeutic potential of cannabinoids. This research has resulted in considerable data related to cancer, albeit most findings being obtained from in vitro experiments. Despite the lack of clinical studies, the potential use of cannabinoids in the treatment of various cancers, such as prostate, breast, and colon cancer, cannot be discounted. There is substantial experimental evidence that supports the positive role that cannabinoids play in cancer cell apoptosis, in preventing metastasis and in the reduction of tumor growth. However, there is not much data available on the pharmacodynamics and pharmacokinetics of cannabinoids. Such studies will provide more information about the dose, route of administration, and in vivo effects when used to treat prostate cancer patients. This will enable us to further explore the unrevealed properties of various cannabinoids, such as the phytocannabinoids, endogenous cannabinoids, and synthetic cannabinoids that may be responsible for the anti-cancer effect. With such knowledge, cannabinoids could become a therapy of choice in the contemporary oncological treatment of prostate cancers.

## 4. Material and Methods

### 4.1. Search Strategy

We searched the PubMed, Embase, Scopus, and Web of Science electronic databases for all relevant studies that have been published from inception to August 2020. The key terms used were related to cannabis combined with prostate cancer, such as cannabinoids OR cannabis AND ‘prostate cancer’ (details of other search terms and search strategies are presented in supplementary file, Table S1). A manual search of all references and citations from the relevant articles was also performed.

### 4.2. Inclusion Criteria

Articles were included in this review if they met the following criteria: (1) In vivo/xenograft studies that reported a clear association between cannabinoids and cannabinoid receptor (CB1 and CB2) activation and further induced cell cycle arrest and apoptosis; (2) cannabinoids inhibiting tumor growth, proliferation, migration, and invasion in animal models; (3) phytocannabinoids or synthetic cannabinoids as an intervention with any dose or duration and that reported at least one tumor-related outcome; and (4) all study designs except reviews, commentaries, case-studies, and the expert opinions. All clinical and in vitro studies and those that did not use cannabinoids were excluded. All identified studies from electronic databases searched were screened according to the inclusion and exclusion criteria.

### 4.3. Study Selection and Data Extraction

Reference citations were exported to Endnote for the removal of duplicates. Eligible studies were identified after independent screening (N.J. and K.S.) of the titles and abstracts based on eligibility criteria. Any disagreements were resolved after discussion or by a third author. Data were extracted independently by two authors (N.J. and K.S.) after conducting full-text screening with a focus on identifying anti-tumor activities of cannabinoid administration in vivo related to prostate cancer. For each study, data was extracted on the first study author, publication year, study design, animal species/strain used, age, numbers used, tumor induction (stating the cell line used), cannabinoid intervention(s), dose size, duration of the study, and relevant outcomes.

### 4.4. Risk of Bias Assessment

Two authors (N.J. and K.S.) independently assessed the risk of bias for each study included in this review. The assessment was performed according to SYRCLE’s ROB tool [49]. We assessed the risk of bias for random group allocation, baseline group characteristics, allocation concealment, random housing, the blinding of examiners and assessors, and random outcome selection. The risk of bias due to dropout/attrition (column#8, Table 1) was not assessed as it was not reported in any study. To assess whether studies were free of other risks of bias, aspects such as increasing the number of animals in groups while conducting the experiment and any possible conflicts of interest were accounted for while reviewing the included studies. We also assessed reporting of the following study quality indicators: Blinding at any level; any randomization; sample size calculation; and temperature control (see Table 1 and Figure 2).

## 5. Conclusions

In summary, cannabinoids were shown to reduce the size of prostate tumors in mice and as such, it can be concluded that they possess anticancer properties. As for their effectiveness, it is not possible to evaluate this, as it depends on the cannabinoid itself or combinations thereof that are used for treatment, as both synthetic and natural cannabinoids have shown anti-cancer outcomes. As reported by Baram [44], not all THC-rich cannabis extracts have the same effect on a particular cell line at a similar concentration. Other compounds that are present in total cannabis extracts besides THC and CBD need to be identified and analyzed to understand their efficacy as anti-tumor agents. Further in vitro and in vivo analyses are required to identify which cannabinoid compounds are better and in what combination. Therefore, further controlled and longer-duration animal studies are warranted to quantify these findings.

## Figures and Tables

**Table 1:** Assessment of the risk of bias in animal studies using SYstematic Review Centre for Laboratory animal Experimentation (SYRCLE) *.

First Author (Year) [Reference]	Selection Bias			Performance Bias		Detection Bias		Attrition Bias (Drop-Outs)	Other Biases			
	Random Group Allocation	Baseline Group Characteristic	Allocation Concealed	Random Housing	Blinding of Examiners	Random Outcome Selection	Blinding of Assessor		Any Randomization	Any Blinding	Size Calculation	Temp Control
Morell (2016) [33]	L	H	H	?	?	H	?	?	Y	N	N	Y
De Petrocellis (2013) [45]	?	H	H	?	H	H	?	?	N	N	N	Y
Roberto (2019) [46]	L	H	?	?	?	?	?	?	Y	N	N	N
Olea-Herrero (2009) [50]	?	L	?	?	?	?	?	?	N	N	N	N
Mukhtar (2007) [51]	?	H	?	?	?	?	?	?	N	N	N	N
Morales (2013) [52]	?	H	?	?	?	H	?	?	N	N	N	N

* H = high risk of bias; L = low risk of bias; ? = unclear; Y = yes reported; N = not reported.

**Table 2:** Characteristics of the in vivo identified studies. All of these studies were performed in male animal models.

First Author (Year) [Reference]	Study Population (Animals)			Tumor Induction (Cell Line)	Study Intervention		Cannabinoid Dose (Route)	Duration	Anticancer Outcomes
	Strain	Age	Number		Intervention	Control			
Roberto, 2019 [46]	Male athymic nu/nu mice	6 weeks	10 mice (*n* = 5 per group)	Xenograft PC-3 cells, DU145 cells, and LNCaP cells	WIN55,212-2	Vehicle (DMSO)	For PC3 and DU145 cell lines, dose given was 5, 10, and 20 µM. For LNCaP cell line, dose given was 20 and 30 µM.	38 days	In PC3 xenograft, dose of 5 µM = 50%, 10 µM = 55%, and 20 µM = 64% reduction in cell proliferation. In DU145 xenograft, dose of 5 µM = 46%, 10 µM = 51%, and 20 µM = 65% reduction in cell proliferation. In LNCaP xenograft, dose of 20 µM = 69% and 30 µM = 66% reduction in cell proliferation.
Morell, 2016 [33]	Athymic nude-Foxn1 (nu/nu)	4 weeks	8 mice	Xenograft PC3 cells	WIN55,212-2	Vehicle (not mentioned)	Daily (i.p.) 0.5 mg/kg (s.c.)	15 days	WIN55,212-2-treated xenografts grew slower and the size of the tumor was smaller than that of vehicle-treated xenografts (% reduction in tumor size not mentioned). βIII Tub levels decreased in WIN55,212-2-treated tumors.
De Petrocellis, 2013 [45]	MF-1 nude mice	4–7 weeks	60 mice (*n* = 10 per group)	Xenograft LNCaP cells	Control CBD-BDS alone	Vehicle (not mentioned)	Daily (i.p.)	35 days	CBD-BDS dose-dependently inhibited the growth of xenografts from LNCaP, but not DU145, cells. At 100 mg/kg, extract exerted a similar effect on both LNCaP and DU145 CBD-BDS plus bicalutamide significantly prolonged survival compared with bicalutamide or CBD-BDS alone.
							Grp 1—vehicle only Grp 2—1 mg/kg daily Grp 2—10 mg/kg daily Grp 2—100 mg/kg daily		
					Docetaxel Bicalutamide CBD-BDS + Docetaxel CBD-BDS + Bicalutamide		Grp 3—5 mg/kg (i.v.) 1× week Grp 4—25–50 mg/kg 3× week (p.o.) Grp 5—100 mg/kg (i.p.) + 5 mg/kg (i.v.) 1× week Grp 6—100 mg/kg (i.p.) + 25–50 mg/kg (p.o.) 3× week		
	MF-1 nude mice	4–7 weeks	60 mice (*n* = 10 per group)	Xenograft DU145 cells	Control CBD-BDS alone	Vehicle (not mentioned)	Daily (i.p.)	35 days	Tumor growth potentiation CBD-BDS + Docetaxel (exact % of reduction not mentioned). CBD-BDS + bicalutamide at 25 mg/kg significantly inhibited xenograft growth (exact % of reduction not mentioned). CBD-BDS + bicalutamide significantly prolonged survival compared with bicalutamide or CBD-BDS alone.
							Grp 1—vehicle only Grp 2—1 mg/kg daily Grp 2—10 mg/kg daily Grp 2—100 mg/kg daily		
					Docetaxel Bicalutamide CBD-BDS + Docetaxel CBD-BDS + Bicalutamide		Grp 3—5 mg/kg (i.v.) 1× week Grp 4—25–50 mg/kg 3× week (p.o.) Grp 5—100 mg/kg (i.p.) + 5 mg/kg (i.v.) 1× week Grp 6—100 mg/kg (i.p.) + 25–50 mg/kg (p.o.) 3× week		
Morales, 2013 [52]	Athymic nu/nu mice (BALB/cOlaHsd-Foxn1nu)	5 weeks	16 mice (*n* = 8 per group)	Xenograft LNCaP cells	PM49 (synthetic cannabinoid quinone)	Control (vehicle not mentioned)	2 mg/kg (i.p)	15 days	Treatment with PM49 almost totally blocked the growth of LNCaP tumors.
				Xenograft PC3 cells					40% tumor growth inhibition and final tumor volume was smaller in all four treated mice.
Olea-Herrero, 2009 [50]	Athymic nu/nu mice	6 weeks	24 mice (*n* = 8 per group)	Xenograft PC3 cells	JWH-015	Control (saline)	1.5 mg/mL (s.c.)	14 days	Final tumor volume and tumor weight were significantly lower in the treatment group (exact % of reduction not mentioned).
							1.5 mg/mL (s.c.)		
					JWH-015 + SR2		1.5 mg/mL + 1.5 mg/kg (s.c.)		
Mukhtar, 2007 [51]	Athymic nu/nu mice	6–8 weeks	24 mice (*n* = 8 per group)	Xenograft 22Rν1 cells	WIN55,212-2	Control	0.5 mg/kg (i.p) alternate day	35 days	Inhibition of tumor growth and decrease in Serum PSA levels to 1.86 ng/mL, whereas that of control group was 7.1 ng/mL. PSA secretion was correlated with tumor growth inhibition (exact % of reduction not mentioned).

i.p. = intraperitoneal; S.C. = subcutaneous; i.v. = intravenous; p.o. = per os (oral administration); PSA = prostate-specific antigen; CBD-BDS = cannabidiol-botanical drug substance.

## Supplementary Materials

The following are available online at https://www.mdpi.com/1422-0067/21/17/6265/s1.
