# Supplementary material for: Cannabinoids and Prostate Cancer: A Systematic Review of Animal Studies
Source: Int J Mol Sci. 2020 Aug 29;21(17):6265. doi: 10.3390/ijms21176265 (PMC7503992; doi:10.3390/ijms21176265)
Supplement: Supplementary file 1 [file ijms-21-06265-s001.pdf]

Supplementary data

Table S1. Search terms and Search Strategy documentation.

Databases searched	Key terms	Search terms used
**PubMed EMBASE SCOPUS Web of Science**	Cannabis or cannabis-related	cannabinoids OR cannabis OR “*Cannabis sativa*" OR Tetrahydrocannabinol OR THC OR cannabidiol OR anandamide OR endocannabinoids OR 2-AG OR 2-Arachidonoylglycerol
	Synthetic cannabinoids	HU-210 OR “WIN-55” OR "212-2" OR “WIN-55,212-2” OR "methanandamide" OR "JWH-015"
	Prostate cancer	Prostatic Neoplasms [MeSH] OR “prostate cancer” OR prostate
	Experimental	*in vivo* OR *ex vivo* OR animal OR experimental OR xenograft

PubMed strategy which was adapted for other databases:

1. cannabinoids OR cannabis OR “*Cannabis sativa*" OR Tetrahydrocannabinol OR THC OR cannabidiol OR anandamide OR endocannabinoids OR 2-AG OR 2-Arachidonoylglycerol
2. HU-210 OR “WIN-55” OR "212-2" OR “WIN-55,212-2” OR "methanandamide" OR "JWH-015"
3. #1 OR #2
4. Prostatic Neoplasms [MeSH] OR “prostate cancer” OR prostate
5. #3 AND #4
6. *in vivo* OR *ex vivo* OR animal OR experimental OR xenograft
7. #3 AND # 6
8. #5 AND #7
